# Virtual surgical planning in orthognathic surgery with the use of patient-specific plates compared with conventional plates. A systematic review focusing on complications, financial expenses, professional and patient-reported outcome measures

**DOI:** 10.4317/medoral.25424

**Published:** 2022-09-29

**Authors:** Özlem Kesmez, Adaia Valls-Ontañón, Thomas Starch-Jensen, Orion Luiz Haas-Junior, Federico Hernández-Alfaro

**Affiliations:** 1 Department of Oral and Maxillofacial Surgery, Aalborg University Hospital, Aalborg, Denmark; 2 Institute of Maxillofacial Surgery, Teknon Medical Centre Barcelona, Barcelona, Spain; 3 Department of Oral and Maxillofacial Surgery, Universitat Internacional de Catalunya, Sant Cugat del Valle`s, Barcelona, Spain; 4 Department of Oral and Maxillofacial Surgery, Pontifical Catholic University of Rio Grande do Sul – PUC/RS, Rio Grande do Sul, Brazil

## Abstract

**Background:**

Orthognathic surgery is a well-known surgical procedure for correction of facial deformities. The surgical procedure is performed by the use of conventional plates and by patient-specific osteosynthesis plates (PSOPs). The aim of this study is to investigate any differences in complications, financial expenses, professional and patient-reported outcome measures (PROM) in orthognathic surgery performed by conventional plates and by PSOPs.

**Material and Methods:**

A MEDLINE (PubMed), Embase, and Cochrane Library search was conducted. Human studies published in English through August 27, 2020 were included. Grey literature, unpublished literature as well as other databases like Scopus, Google Scholar, or Research Gate were also included in the search strategy of the present systematic review. Randomized and controlled clinical trials were included. Risk of bias was assessed by Cochrane risk of bias tool and Newcastle-Ottawa Scale.

**Results:**

Five studies with unclear risk of bias and moderate quality were included. Meta-analysis was not applicable due to considerable heterogeneity. There was no significant difference in intra- and postoperative complications or professional and PROM with the two treatment modalities, although higher tendencies to reoperations were observed with conventional plates. Financial expenses were significantly higher with PSOP, but treatment planning and intraoperative time were shortened by approximately one third compared with mock surgery and conventional plates.

**Conclusions:**

No significant differences were observed in complications, professional and PROM. Higher financial expenses were recorded in orthognathic surgery performed with PSOP. Treatment planning and intraoperative time were shortened with the use of conventional plates. Although further randomized trials are needed before definite conclusions can be provided about beneficial use of PSOPs in orthognathic surgery from a professional and patient perspective.

** Key words:**Orthognathic surgery, systematic review, virtual planning.

## Introduction

PredicTable transmission of the treatment plan and accurate intraoperative repositioning of the bone segments are essential to obtain optimal aesthetic and functional outcome in orthognathic surgery (1-2). Traditional preoperative treatment planning of dentofacial deformities involves reproduction of the occlusal discrepancy on a semi-adjusTable articulator through facebow transfer of cast model, two-dimensional cephalometric analysis, mock surgery, and manual fabricated acrylic occlusal splints (3-5). However, this treatment planning approach contain potential errors and inaccuracies related to the impression, facebow transfer, simulation of the surgical plan, and intraoperative repositioning of the bone segments (6-8). Cone beam computed tomography (CBCT) and computer-assisted technologies enable acquisition of three-dimensional images of the craniofacial complex and fabrication of computer-generated occlusal splints. Novel treatment strategies have therefore been explored to improve the accuracy in orthognathic surgery including three-dimensional virtual planning, surgical guided navigation, customized surgical drill guides, and milled or laser sintered patient-specific osteosynthesis plates (PSOP) (1-5). Three-dimensional virtual surgical planning with individually designed customized surgical drill guides and PSOP poses several advantages including surgical guide-oriented osteotomies, obviate intraoperative measurements, accurate three-dimensional repositioning of the bone segments without an occlusal splint, elimination of intraoperative plate bending, precise placement of screws, and shortened time in the operating theatre (1-5). Previous systematic reviews have demonstrated comparable or higher accuracy with the use of three-dimensional virtual planning involving PSOP compared with two-dimensional planning and conventional plates in orthognathic surgery (4). However, the use of PSOP are claimed to be associated with higher cost, longer treatment planning time since designing and manufacturing of patient-specific supportive materials are time-consuming, risk of screw placement in fragile maxillary bone, and inability of modifying the surgical plan intraoperatively as the treatment are predetermined by the PSOP (9-11). Moreover, the conFiguration and design of PSOP are often bulky and rough, which requires a wider surgical field, which needs further subperiosteal dissection and consequently increases the risk of contamination, infection, wound dehiscence, and postoperative plate exposure (12). Consequently, these disadvantages seem to restrict routine use of PSOP in orthognathic surgery. On the contrary, reduced time in the operating theatre could potentially lead to a better outcome for the patient including diminished blood loss and shortened hospitalization, which could justify the additional preoperative costs that come with three-dimensional virtual surgical planning and individually designed PSOP (13-14). Thus, the objective of the present systematic review is to test the hypothesis of no difference in complications, financial expenses, professional and patient-reported outcome measures (PROM) following virtual surgical planning in orthognathic surgery with PSOP compared with conventional plates.

## Material and Methods

- Protocol and registration

**Figure 1:**
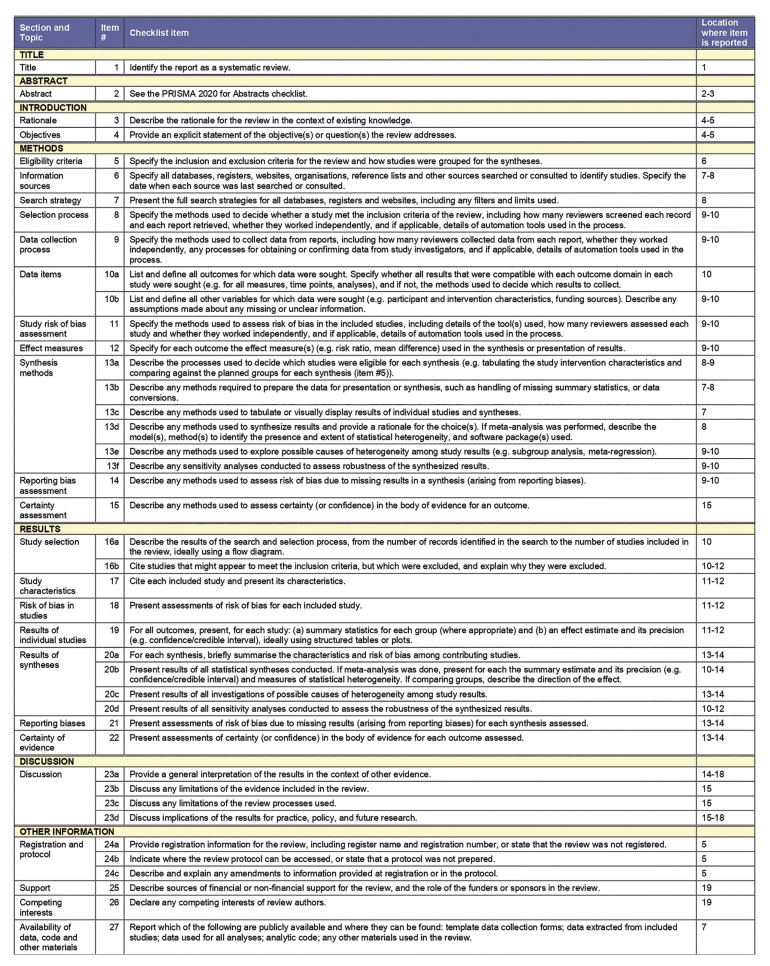
PRISMA checklist.

Review was conducted in accordance with the Preferred Reporting Items for Systematic reviews and Meta-Analyses (PRISMA) statement for reporting systematic reviews (15). The PRISMA checklist is illustrated in Fig. 1. Methods of the analysis and inclusion criteria were specified in advance and documented in a protocol and registered in PROSPERO, an international prospective register of systematic reviews.

Registration number: CRD42020207539.

The protocol can be accessed at:

https://www.crd.york.ac.uk/prospero/display_record.php?ID=CRD42020207539.

- Focus question

Focus question was developed according to the Patient, Intervention, Comparison and Outcome (PICO) framework as described in Table 1.

- Study design eligibility criteria

Randomized controlled trials and controlled trials in humans assessing complications, financial expenses, professional and PROM following virtual planning in orthognathic surgery with the use of PSOP compared with conventional plates.

**Table 1:**
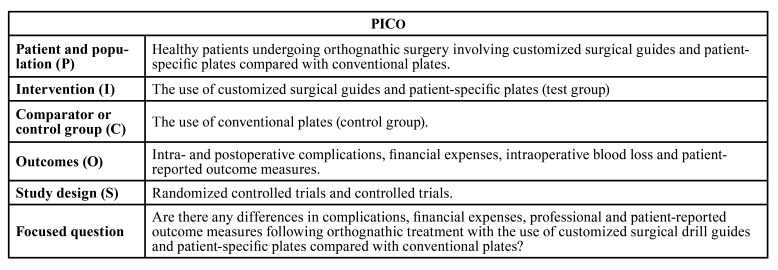
PICOS criteria.

- Types of outcome measures

1) Intra- and postoperative complications including failures related to the patient-specific material such as improper fixation, misfit, breakage or deformation as well as dental and periodontal injuries, infection, mucosal dehiscence, soft tissue problems, bone necrosis, non-union of bone segments, limited mouth opening, fixation material failure, removal of screw and plates, maxillary sinusitis, severe swelling, fistula, and reoperation with the two treatment modalities.

2) Financial expenses including cost-effectiveness as well as length of treatment planning, time in the operating theatre, and hospitalization.

3) Intraoperative blood loss.

4) PROM as evaluated by interview, questionnaire, and visual analogue scale.

5) Professional assessment as evaluated by surgeon’s satisfaction with handling of the PSOP or the surgical outcome.

- Information sources

The search strategy incorporated examinations of electronic databases, supplemented by a thorough hand-search page by page of relevant journals including “British Journal of Oral and Maxillofacial Surgery”, “International Journal of Oral and Maxillofacial Surgery”, “Journal of Dental Research”, “Journal of Oral & Maxillofacial Research”, “Journal of Craniofacial Surgery”, “Journal of Cranio-Maxillo-Facial Surgery”, “Journal of Oral and Maxillofacial Surgery”, “Oral and Maxillofacial Surgery” and “Oral Surgery Oral Medicine Oral Pathology Oral Radiology”. The manual search also included bibliographies of all articles selected for full-text screening as well as previously published reviews. Two reviewers (ÖK, TSJ) independently performed the search. In the event of disagreement between the reviewers, another reviewer was consulted (AVO).

- Search strategy for identification of studies

A MEDLINE (PubMed), Embase, Cochrane Library search was conducted. Human studies published in English through August 27, 2020 were included. Grey literature, unpublished literature as well as other databases like Scopus, Google Scholar, or Research Gate were also included in the search strategy of the present systematic review. The search strategy was performed in collaboration with a librarian and utilized a combination of Medical subject heading (MeSH) and free text terms.

- Selection of studies

**Figure 2:**
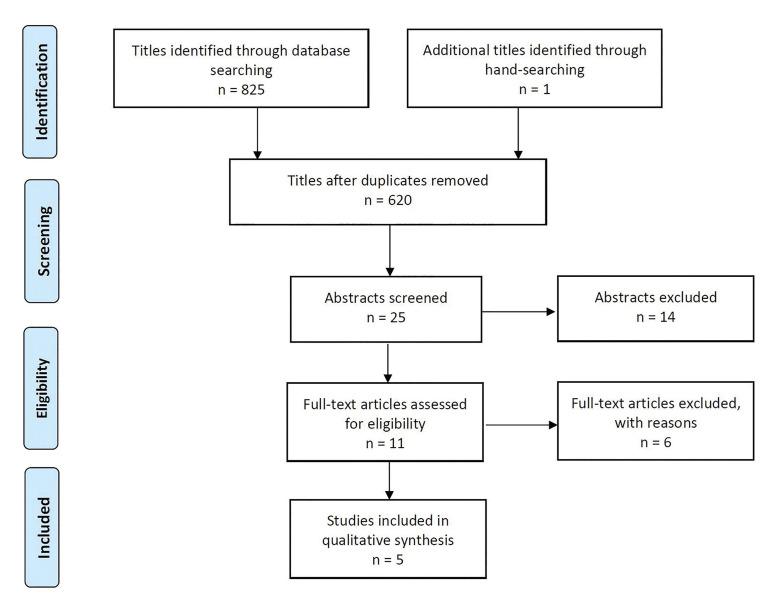
PRISMA (Preferred Reporting Items for Systematic Reviews and Meta-Analyses) flow diagram demonstrating the first hit retrieved a total of 825 records. The distribution of the searched records and the number of studies finally selected are shown in the flow diagram.Cochrane Collaboration´s tool for assessing risk of bias for randomized controlled trials.

In PubMed, Embase, Cochrane Library, and web of science a total of 825 titles were identified by the 27th of August 2020. After duplicate removal using EndNote a total of 620 titles were identified. PRISMA flow diagram presents an overview of the selection process (Fig. 2). Abstracts were assessed when titles indicated that the study was relevant. Full-text analysis was obtained for those with apparent relevance or when the abstract was unavailable. References of papers identified and previously published systematic reviews assessing complications, financial expenses, professional and PROM were cross-checked for unidentified articles. Study selection was performed by two reviewers (ÖK and TSJ). In the event of disagreement between the reviewers, another reviewer was consulted (AVO). The level of agreement between the authors was tested using Cohen’s kappa coefficient (k).

- Inclusion criteria

Human studies assessing three-dimensional virtual planning and computer-assisted technologies in orthognathic surgery with the use of PSOP compared with conventional plates in conjunction with two- or three-dimensional planning were included, if they reported the previously described outcome measures. In addition, at least five patients had to be included in the study and the surgical procedure should be clearly specified.

- Exclusion criteria

Following exclusion criteria were applied: letters, editorials, PhD theses, letters to the editor, case reports, abstracts, technical reports, conference proceedings, cadaveric studies, animal or *in vitro* studies, and literature review papers were excluded. Moreover, studies using prebent osteosynthesis plates or wires as internal fixation method were also excluded.

- Data extraction

Data were extracted by one reviewer (TSJ) according to a data-collection form ensuring systematic recording of the outcome measures. In addition, relevant characteristics of the study were recorded. Corresponding authors were contacted by e-mail in the absence of important information or ambiguities.

- Data items

Following items were collected and arranged in following fields: source, study design, number of patients, surgical procedure, material, observation period, complications, financial expenses, professional and PROM.

- Quality and risk-of-bias assessment

Quality assessment was undertaken by one review author (TSJ) as part of the data extraction process. Cochrane Collaboration’s tool for assessing risk of bias suggested in the Cochrane Handbook for Systematic Reviews of Interventions was used for included randomized controlled trials (version 5.1.0) (16). Following items were evaluated: random sequence generation, allocation concealment, patient blinding, outcome blinding, incomplete outcome data addressed and selective reporting. Publications were grouped into the following categories: low risk of bias (possible bias not seriously affecting results) if all criteria were met; high risk of bias (possible bias seriously weakening reliability of results) if one or more criteria were not met; or unclear risk of bias when too few details were available for classification as high or low risk.

Newcastle-Ottawa scale (http://www.ohri.ca/programs/clinical_epidemiology/oxford.asp) was applied for non-randomized studies. Following items were evaluated: selection of studies, comparability of cohorts, and ascertainment of either the exposure or outcome of interest.

Stars were awarded with highest quality studies awarded up to nine stars. Included non-randomized studies were categorized as: low-quality (0 - 3 stars), moderate quality (4 - 6 stars) or high quality (7 - 9 stars).

## Results

- Study selection

Search results are outlined in Fig. 2. Electronic search resulted in 825 entries. One article was identified through hand-searching. Of these 826 articles, 206 were excluded due to being retrieved in more than one search. A total of 25 abstracts were reviewed and full-text analysis included 11 articles. Finally, five comparative clinical trials were included (17-21).

The level of agreement between the two authors (ÖK and TSJ) in selecting studies to be read in full was measured at k = 0.682 (95% confidence interval: 0.501-0.863), while level of agreement between the two authors (ÖK and TSJ) for eligibility assessment was measured at k = 1.00.

- Exclusion of studies

Reasons for excluding six studies after full-text assessment were: none of the outcome measures were reported (n = 1) (22), prebent osteosynthesis plates were used (23-24), PSOP were not used (25-26), and case-serie (27).

- Characteristics of the studies included

Three randomized controlled trials (18,20-21), and two controlled trials were included in the present systematic review (17,19). Randomization was conducted by computer software (18,20), or blocked randomization using the Sealed Envelope online tool (21). Power calculation of the sample size was performed in one study (21). Age and gender distribution were clearly specified in all the included studies (17-21). Defined inclusion and exclusion criteria were clearly described in three studies (28,22-21). Image acquisition, three-dimensional virtual planning, surgical simulation, as well as designing and manufacturing of PSOP was performed using dissimilar software systems including Planmeca ProModel System (Planmeca Ltd, Helsinki, Finland) (17,19), Mimics 19.0 (Materialise NV, Leuven, Belgium) (20), 3-matic 11.0 (Materialise NV, Leuven, Belgium) (20), Maxilim v2.3 (Medicim NV, Mechelen, Belgium) (21), or no information was provided about the used software program (18). Customized surgical drill guides were manufactured in white polyamide using fused deposition modelling technology (20), three-dimensional printing in resin-based material (21), or no information was provided about the used technique (17-19). PSOP was manufactured in titanium using laser sintering (20), milling (19,21), or no information was provided about the used technique. (17-18). Conventional plates included MatrixORTHOGNATHIC (DePuy Synthes, Oberdorf, Switzerland) (17-19), or no information was provided about the brand (18,20-21). The intermediate acrylic resin splint fabricated on a semi-adjusTable articulator (17-18,20). The surgical procedure was planned and performed by one surgeon (17), two surgeons (21), or by an unknown number of surgeons (18-20). Experience of the surgeon was not described in any of the included studies. The maxilla-first approach without a splint for maxillary positioning was used in three studies (17,20-21), while no information was provided about the surgical sequence or use of splint (18). In bilateral sagittal split osteotomy or bimaxillary surgery, positioning of the mandible was guided by a computer-assisted printed splint (19,21), or an acrylic splint fabricated on a hinge articulator (20). Blinded assessment was conducted in one study (20). Complications were assessed by clinical examination (17). PROM were reported by self-administrated orthognathic quality of life questionnaire (18), or no information’s was provided about the assessment method (20). Information about drops-out was reported in one study (21). Methods for examiner training or calibration was not reported in any of the included studies.

- Data synthesis

Meta-analysis can only be conducted for continuous data if both the mean and standard deviation are available for similar comparison with identical outcome measures. However, the included studies of the present systematic review solely reported mean values with or without standard deviation. Thus, meta-analysis was not applicable.

- Methodological quality

Quality of the included studies is summarized in Table 2 and Table 3.

**Table 2:**
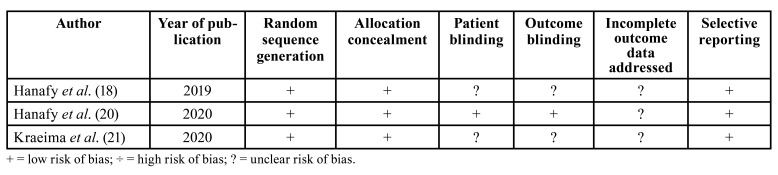
Cochrane Collaboration´s tool for assessing risk of bias for randomized controlled trials.

**Table 3:**
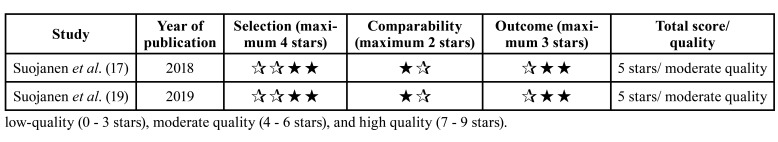
Newcastle-Ottawa scale for assessing quality of non-randomized studies.

- Outcome measures

Results of each outcome measures are presented below and outlined in Table 4. Amount of intraoperative blood loss was not reported in any of the included studies. Hence, this outcome measure is not described. Reported numerical values are presented as mean values with standard deviation.

**Table 4:**
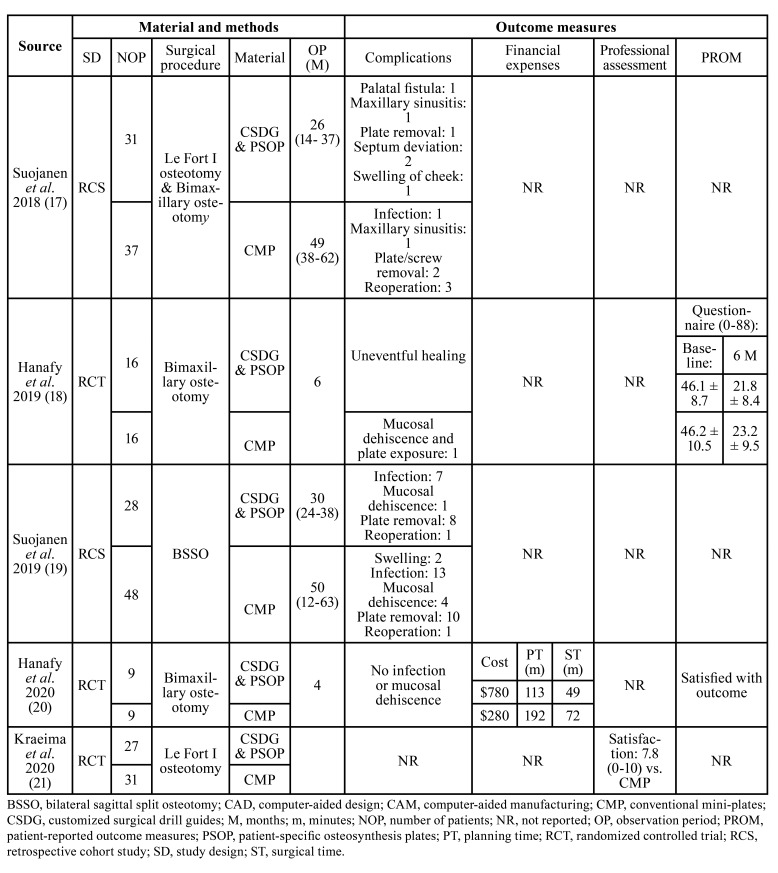
Intra- and postoperative complications, financial expenses, professional and patient-reported outcome measures.

- Complications

The frequency of intra- and postoperative complications was reported in four studies (17-20). Infection, mucosal dehiscence, and plate exposure were the most commonly reported complications. There was no significant difference between the two treatment modalities, although there seems to be a higher tendency for reoperation with conventional plates compared with PSOP (17).

- Financial expenses

Financial expenses were reported in one study (20). The approximated cost was significantly higher with customized surgical drill guides and PSOP (USD $780) compared with conventional plates (USD $280) (20). However, the treatment planning time from the end of the virtual plan to export of the stereolithography file was shortened with computer-aided surgery (113 minutes) compared with mock surgery on a semi-adjusTable articulator using facebow record (192 minutes). Moreover, the intraoperative time from maxillary incision to fixation was shortened with customized surgical drill guides an PSOP (49 minutes) compared with conventional plates (72 minutes) (20). The actual financial expenses therefore seem to be increased with the use of customized surgical drill guides and PSOP compared with conventional plates, but treatment planning time as well as intraoperative time were shortened by approximately one third.

- Professional reported outcome measures

Professional reported outcome measures were reported in one study (21). The surgeon’s overall satisfaction with the user-friendliness as well as the position of the maxilla was 7.8 on a scale from zero to ten with customized surgical drill guides and PSOP compared with previous experience using conventional plates (21). The drilled screw holes [8.1], screw placement [8.1], position of the maxilla [8.4], placement of the guide [7.5], and indication of screw holes [7.1] were also evaluated (20).

- Patient-reported outcome measures

PROM were reported in two studies (18,20). Verbal response and self-administrated orthognathic quality of life questionnaire revealed no significantly differences between the two treatment modalities (18,20). The overall baseline score containing four domains (facial aesthetics, oral function, awareness of deformity, and social aspect) decreases from 46.1 (SD: 8.7) to 21.8 (SD: 8.4) on a scale ranging from zero to 88 with customized surgical drill guides and PSOP (*P* < 0.001), after six months. Corresponding measurements with conventional plates were 46.2 (SD: 10.5) to 23.2 (SD: 9.5) (*P* < 0.001) (18).

## Discussion

The objective of the present systematic review was to test the hypothesis of no difference in complications, financial expenses, professional and PROM following virtual surgical planning in orthognathic surgery with the use of PSOP compared with conventional plates. Three randomized controlled trials with unclear risk of bias and two controlled trials of moderate quality were included in the present systematic review (17-21). There was no significant difference in the frequency of intra- and postoperative complications or professional and PROM with the two treatment modalities, although a higher tendency to reoperations were reported with the use of conventional plates. The financial expenses were significantly higher with customized surgical drill guides and PSOP, but treatment planning time and intraoperative time were shortened by approximately one third compared with mock surgery on a semi-adjusTable articulator using facebow record and conventional plates (20). Consequently, customized surgical drill guides and POSP are currently associated with higher cost, but the shortened treatment planning and time in operating theatre may compensate for the higher financial expenses.

The strength of evidence from a systematic review and meta-analysis is related to the quality of the included studies. Quality and risk-of-bias assessment revealed some methodological confounding factors among the included studies of the present systematic review and considerable heterogeneity prevented meta-analysis from being performed. The current level of evidence is therefore inadequate to propose specified implications for evidence based clinical guidelines according to the focus question of the present systematic review. Further randomized controlled trials with larger patient samples assessing accurate transmission of the treatment plan, complications, economic perspectives as well as professional and PROM with the two treatment modalities are therefore needed before definite conclusions can be provided about the beneficial use of customized surgical drill guides and PSOP in orthognathic surgery according to the focus question of the present systematic review.

Intra- and postoperative complications are unanticipated or unfavourable outcome of a treatment. Errors related to the design and manufacturing of customized surgical drill guides and PSOP may cause intraoperative misfit, improper fixation, or deformation, which adversely affect the transmission of the treatment plan and accurate reposition of the bone segments (21,28-29). Conventional occlusal splints are therefore occasionally manufactured as a safety precaution, if the patient-specific material cannot be used (21). In the present systematic review, conversion of the treatment strategy from customized surgical drill guides and PSOP to conventional plates was reported in one study due to damaged or incomplete customized surgical drill guides or PSOP after sterilization, late change in the surgical planning, and intraoperative conversion to the control group (21). These necessary safety precautions in conjunction with the use of customized surgical drill guides and PSOP constitute a significant drawback due to prolonged treatment planning time, higher cost and risk of surgical inaccuracies. Moreover, a newly published study described increased caution and counselling with utilization of patient-specific mandibular implants in patients with syndromic status, history of orofacial cleft, and history of previous maxillomandibular surgery due to increased risk of hardware-related complications (30). These results are in accordance with a previous study assessing bilateral sagittal split osteotomy with customized surgical drill guides and PSOP reporting total precision of the patient-specific material in solely 11 out of 30 patients (28). Further development and improvement in the manufacture of patient-specific material is therefore needed to ensure satisfying durability, strength, and accuracy in orthognathic surgery. Thus, surgical splints printing is still required.

Infection, mucosal dehiscence, severe swelling, dental and periodontal injuries, bone necrosis are well-known complications in orthognathic surgery (31-32). The prevalence of complications in the included studies of the present systematic review seems to be comparable with previous studies (30). Risk of infection, mucosal dehiscence, and soft tissue problems are commonly related to age, gender, smoking habits, duration of surgery, surgeon experience, surgical site, previous surgeries, and type of osteotomy performed (33-34). Description of duration of surgery, surgeons experience nor correlation analysis of relationship between age or gender and percentage of complications have not been performed in any of the included studies of the present systematic review.

Estimating the total financial expenses associated with a specific treatment modality is difficult to compare since the cost attribuTable the national health care system and dissimilar calculation methods. Operating theatres generally account for a large percentage of a hospital's total revenue and improving operating theatre efficiency can considerably affect the overall cost and improve health care outcome. In the present systematic review, the cost of customized surgical drill guides and PSOP in Egypt was significantly higher compared with conventional plates, but treatment planning time and time in the operating theatre were shortened by approximately one third compared with mock surgery on a semi-adjusTable articulator using facebow record and conventional plates (20). Recent studies have reported lower cost and shorter treatment planning time with three-dimensional virtual planning and manufacturing of occlusal splints by computed technologies compared with conventional treatment planning and manual splint fabrication, although customized surgical drill guides and PSOP were not used in any of these studies (9-11,26). Patient-specific material is often manufactured in an outsourced laboratory, which significantly increases the cost. Development of low-cost technologies as well as in-house three-dimensional printer or milling device will efficiency improve the cost-effectiveness and accuracy of the process by reducing errors and extra charges from the outsourced laboratory. Furthermore, a newly published study concluded that computed technology for mandibular reconstruction will become a widely used reconstructive method and that its cost will be covered by gains in terms of surgical time, quality of reconstruction, and reduced complications (35).

Reduced length of hospitalization may decrease risk of infection, medication side effects, improvement in the quality of treatment, and increased hospital profit with more efficient bed management (36). A previous study has shown a significant correlation between duration of surgery and length of hospitalization in orthognathic surgery (33), which is in accordance with the conclusions of a newly published systematic review and meta-analysis (3). In the present systematic review, time in the operating theatre were shortened by approximately one third with the use of customized surgical drill guides and PSOP compared with conventional plates (20). Consequently, customized surgical drill guides and PSOP in orthognathic surgery will shortened time in the operating theatre, which may possibly diminish risk of complications, reduce financial expenses, and shortened hospitalization.

PROM are commonly used in orthognathic surgery to assess patients' perception of the treatment outcome and views of their health status (37-39). Patient expectations and satisfaction following orthognathic surgery are generally high revealing improvement in oral-health related quality of life, psychosocial components, and facial aesthetics (37-39). However, each individual patient has different motivations and expectations, which necessitates standardized, validated, and reliable tools for assessment of PROM in orthognathic surgery (40). In the present systematic review, verbal response and self-administrated orthognathic quality of life questionnaire revealed no significantly differences between customized surgical drill guides and PSOP compared with conventional plates in orthognathic surgery (18,20).

## Conclusions

The hypothesis of no difference in complications, financial expenses, professional and PROM following virtual surgical planning in orthognathic surgery with customized surgical drill guides and PSOP compared with conventional plates is rejected. No significant difference in intra- and postoperative complications or professional and PROM were revealed, though higher tendencies to reoperations were observed with conventional plates. Financial expenses were significantly higher with customized surgical drill guides and PSOP, but treatment planning and intraoperative time were shortened by approximately one third compared with mock surgery on semi-adjusTable articulator using facebow record and conventional plates. Conclusions drawn from results of this systematic review should be interpreted with caution due to dissimilar evaluation methods and various methodological confounding factors among the included studies. Further randomized trials are therefore needed before definite conclusions can be provided about beneficial use of customized surgical drill guides and PSOP in orthognathic surgery according to the focus question of the present systematic review.
